# Notes and News

**Published:** 1895-11-02

**Authors:** 


### Notes and News.

(Collected and Communicated.)

A new isolation hospital has just been opened at Plymouth by the Mayor of that town.

The North London Hospital for Consumption has received a cheque for £1,000, being the second sum of similar amount given to the institution by Mr. Henry Harben.

Last Saturday Mr. Passmore Edwards laid the foundation stone of a much-needed cottage hospital, which is to be established at Tilbury Dock. Mr. Passmore Edwards provides £2,000, whilst the Docks Committee contribute £500 and a site. The public are asked to subscribe the furniture and provide for future maintenance. It is proposed to attach a district nurse and a midwife to the hospital.

The hospitals of Liverpool are sharing some of the recent good fortune which has been shining on the metropolitan charities. Mr. Bernard Vincent Hall has bequeathed no less than £11,000 to be divided between the Royal Infirmary, Northern Hospital, Royal Southern, and Ladies' Charity and Lying-in Hospitals, in varying amounts, the Royal Infirmary heading the list with £5,000.

The new buildings at the Bradford Infirmary, commenced a year ago, were opened last week by the High Sheriff of Yorkshire. A striking feature of the extension is the cooking apparatus, which is on a new plan as far as English institutions are concerned, the whole being arranged in the centre of the kitchen, whereby both time and labour are saved. The cost of the new improvements has been £6,000.

It is announced that a conference of delegates of the London local authorities will be held to consider the position of the Metropolitan Asylums Board as a body which provides for the care of infectious diseases in the metropolis. The point at issue is as to where the responsibility of the local authorities ends in respect to providing for their own fever cases, and what should be definitely required at the hands of the Metropolitan Asylums Board.

On Friday the Westminster Hospital will be formally opened, after having been closed for some time for important improvements. The president, the Duke of Westminster, will attend, and a large number of friends are expected to meet him and inspect the hospital.

Although the petition presented to the Royal College of Physicians praying for the admission of women to its licentiate has been rejected, the majority against the proposal was much less than some had expected. In addition to the ordinary arguments in favour of the equal admission of women to medical qualifications, it is, no doubt, felt that in case the reconstruction of the University of London should involve co-operation with the Royal Colleges for examination purposes, such co-operation would be much facilitated by the various institutions being on an equality in regard to the admission of women.

New regulations are about to be made in Russia in relation to the degree in medicine. Doctors of medicine will in the future be regarded as belonging purely to the scientific grades of professional calling. The degree can only be taken after medical men have held the diploma to practice for three years. Within the five years following the examination the candidate must write a thesis on some professional subject, and if he fails to do this he must pass another examination. The new regulations are destined to make the acquisition of a degree harder than before, and tend towards encouraging specialism, which is carried on to a very great extent in Russia.

The ceremony of laying the foundation stone of a convalescent home for the French Hospital at Brighton acted as a shock to the inhabitants of Brighton. Very few persons appear to have known for what purposes the site at the end of the Kemp Town Parade was sold, and so the brilliant ceremony, in which the French Ambassador took part, acted as a most unpleasant surprise. The objection appears to be rather on the score of manners than of fear of infection of a bodily nature. It is thought that an influx of strangers from the poor neighbourhoods of Leicester Square will not add to the value of property adjacent to the proposed home, and vigorous protest is being made.

Little real progress seems to have been made in settling the question of hospital accommodation for Manchester in relation to the present Royal Infirmary. A meeting of the trustees has been recently held, which shows, however, that amongst their number there are those who recognise the importance of avoiding mistakes which are easily fallen into by those who have not studied the question of hospital building, and the many issues at stake in erecting one. Plans and elevation for buildings on the present site are now to be secured, and the efficiency of such plans is to be submitted to expert opinion, the same expert or experts apparently being required to give an estimate as to cost. A sub-committee is also to be formed for the purpose of investigating the question of the desirability of erecting a supplementary hospital elsewhere, and the cost thereof, and into the best means of bringing the present building up to date. The resolutions thus passed at the meeting appear to open the question up more fully even than before, but any decision appears to be as far off as ever.

